# Multi-Faceted Role of Histone Methyltransferase Enhancer of Zeste 2 (EZH2) in Neuroinflammation and Emerging Targeting Options

**DOI:** 10.3390/biology14070749

**Published:** 2025-06-23

**Authors:** Sotirios Moraitis, Christina Piperi

**Affiliations:** Department of Biological Chemistry, School of Medicine, National and Kapodistrian University of Athens, 11527 Athens, Greece; smoraitis2003@gmail.com

**Keywords:** neuroinflammation, epigenetic, histone methylation, H3K27me3, EZH2 inhibitors, DZNep, EPZ-6438

## Abstract

Neuroinflammation, a complex nervous system response to brain injury and other pathological stimuli, exhibits a common denominator role in the pathogenesis of neurological disorders and their progression. Among several regulators of neuroinflammation, epigenetic mechanisms with particular emphasis on histone methylation have a prominent role by altering the expression of specific genes involved in the onset and progression of neuroinflammation. The Enhancer of Zeste 2 (EZH2) histone lysine methyltransferase is a multi-faceted and context-dependent regulator of immune response and neural cell function, significantly involved in the underlying mechanisms of neuroinflammation, such as inflammatory gene expression, astrocyte function, microglial activation, BBB integrity, and interactions with non-coding RNAs. Herein, we explore the intricate implication of EZH2 activity in the onset of neuroinflammation and associated pathological conditions, and discuss its potential as a therapeutic target. Currently available EZH2 inhibitors with neuroprotective effects are also addressed in an effort to reveal novel strategies for managing neuroinflammatory conditions, and potentially improving neurological health.

## Simple Summary

Neuroinflammation is a common factor underlying the pathogenesis of neurological disorders and their progression. Epigenetic mechanisms can alter the expression of both inflammatory and anti-inflammatory genes involved in the onset and progression of neuroinflammation. The histone methyltransferase EZH2 exerts a multifaceted and context-dependent role in neuroinflammatory processes regulating important parameters such as microglial and astrocyte function, BBB integrity and non-coding RNAs. The targeting of EZH2 at specific times and disease stages can exert neuroprotective effects in neuroinflammation-related diseases.

## 1. Introduction

Neuroinflammation is a complex, inherent protective mechanism employed by the host’s nervous system as a defense mechanism against infectious and pathogenetic conditions. However, it is also a fundamental pathological process underlying a broad range of neurological disorders such as Alzheimer’s disease, Parkinson’s disease, depression, and traumatic brain injury. Acute inflammation underlies the defensive and protective properties of this process, which, after prolonged duration, has a pathogenic outcome. In this case of established chronic inflammation, there is a marked imbalance between anti-inflammatory and pro-inflammatory molecules, such as cytokines, chemokines, growth factors, etc. [1,2]. Resident immune cells of the CNS play a key role in neuroinflammation along with infiltrating peripherally derived immune cells and the presence of multiple inflammatory factors [3,4].

Recently, several epigenetic mechanisms have been associated with the initiation of neuroinflammation [3] and have been understood to contribute to neuroinflammation-related outcomes [5,6]. These mechanisms involve alterations in gene expression without affecting the DNA sequence and primarily include DNA methylation, histone modifications and non-coding RNAs [7]. A predominant role of histone methylation has been observed in inflammatory processes, modulating intracellular signaling in the context of neurological disorders [8,9]. Histone methylation involves methyl groups’ addition to core histone proteins, altering chromatin structure and affecting gene regulation, with a subsequent impact on cell differentiation and function in the CNS [2]. As one of the most prominent and stable post-translational modifiers, histone methylation may induce gene silencing or transcriptional activation, depending on the methylation site, as well as the specific amino acid that is modified [7].

The histone lysine methyltransferase Enhancer of zeste homolog 2 (EZH2), a Polycomb Repressive Complex 2 (PRC2) component, mediates tri-methylation of histone 3 on lysine 27 (H3K27me3), leading to suppressed gene expression. Increasing evidence demonstrates that this histone methyltransferase plays a pivotal role in neuroinflammation-associated neurological diseases through modification of specific genes [10,11], indicating epigenetic regulation as a potential targeting approach to control neuroinflammation [12].

The latest research data showed elevated EZH2 expression in tissues and peripheral blood of individuals affected by diverse neuroinflammation-associated conditions and diseases [10,11,13]. Despite advances in understanding EZH2’s functional role in normal physiology and disease, its impact on neuroinflammatory processes and the underlying mechanisms are still under investigation. This review aims to explore the contribution of EZH2 activity in neuroinflammation, delineate the underlying destructive or protective mechanisms and present potential targeting options for therapeutic intervention in neurological disorders with inflammatory background.

## 2. Structural Aspects of EZH2

EZH2 is a 751-aminoacid histone methyltransferase of about 87 kDa, mapped on chromosome 7q36.1 and the catalytic subunit of PRC2. It encodes five subtypes with type A comprising the most functional one [14].

**Figure 1:**
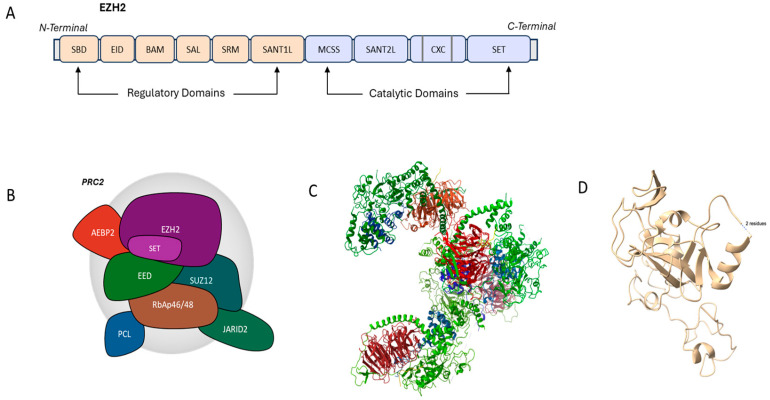
EZH2 gene structure and PRC2 complex components. (**A**) Structure of EZH2 indicating the regulatory (SBD, EID, BAM, SAL, SRM, and SANT1) and catalytic domains (MCSS, SANT2, CXC and SET). (**B**) PRC2 complex composition indicating the core subunits: EZH2 (enhancer of zeste homolog 2), EED (embryonic ectoderm development), SUZ12 (suppressor of zeste 12 homolog), RbAp46/48 (retinoblastoma binding protein 4) and non-core subunits: JARID2 (jumonji, AT-rich interactive domain 2), PCL (polycomb-like proteins), AEBP2 (adipocyte enhancer-binding protein 2). (**C**) Crystal structure of the PRC2 complex with the EZH2 subunit in green, EED in red, SUZ12 in blue, and JARID2 in yellow. (**D**) Two-dimensional structure of EZH2 subunit. The structure was obtained from the Protein Data Bank (PDB ID: [5LS6], [4MI0]), and the image was generated using ChimeraX (version 1.10) [23].

Structurally, EZH2 contains ten discrete domains, namely the SANT1L-binding domain (SBD), the EED-interaction domain (EID), the β-addition motif (BAM), the SET activation loop (SAL), the stimulation-responsive motive (SRM), the SANT1 domain, the motif connecting SANT1L and SANT2L (MCSS), the SANT2 domain, the CXC domain and the SET domain (Figure 1). These domains have diverse structural features and functions and can be categorized as regulatory or catalytic. Among them, the regulatory domains SBD, EID, BAM, SAL, SRM and SANT1 are located near the N-terminal, while the MCSS, SANT2, CXC, and SET domains consist of the catalytic region, near the C-terminal.

The EID domain serves as the binding site to the EED subunit of the PRC2 complex. Histone interacts with SANT1L, while the SUZ12 subunit of the PRC2 complex binds to SANT2L. The interaction of EID and SANT2L with EED and SUZ12, respectively, enables EZH2 to apply its methyltransferase activity [14,15].

At the C-terminus of EZH2 is located the SET domain which maintains the methyltransferase activity since it contains a S-adenosyl methionine (SAM) binding site, enabling the transfer of methyl groups from SAM to histone [14]. Nonetheless, both the C-terminal SET-domain and the contiguous CXC are essential for histone methyltransferase activity [16]. CXC contains a Zn3Cys8His and a ZN3Cys9 cluster and interacts with nucleosomes and DNA [15].

With respect to the spatial structure of EZH2, SRM is adjacent to SET, activates it by signaling, and further supports its configuration by binding to EED. As a result, SRM facilitates the methyltransferase activity of SET [11]. The N-terminal domains are vital for binding with the subunits of the PRC2 complex, providing interaction sites for the assembly with the partner subunits [16,17].

For proper function, EZH2 must interact and bind to the EED and SUZ12 subunits of PRC2 (Figure 1) [18]. The Polycomb Group (PcG) proteins can form complexes capable of modifying chromatin, thus serving as epigenetic regulators. Among them, PRC2 is a principal PcG protein in mammals comprising EED, SUZ12, RbAp46/48, and EZH2 [19]. EED and SUZ12 exhibit a crucial impact on the enzymatic activity of the complex, while RbAp46/48 primarily amplifies the EZH2–EED–SUZ12 interaction.

The recognition of the H3K27me3 mark by EED is a positive feedback mechanism that enables the allosteric activation of the PRC2 enzymatic function [20]. Therefore, the H3K27me3-EED binding is essential for further methylation of any unused H3K27. Another key point is that for the maintenance of the fundamental PRC2 function, the synergy of all EZH2, EED, and SUZ12 is necessary, with the depletion of any of the above utterly dysregulating the PRC2 activity [21]. Additional free proteins can be found in PRC2, including AEBP2, PCL and JARID2 [22].

## 3. Physiological Functions of EZH2

EZH2 is expressed in several cell types and tissues distributed to a wide range of systems, such as the immune, endocrine, muscular and nervous systems [18]. There is evidence of sex-related differences in EZH2 expression and regulation, largely driven by hormonal influences, epigenetic context, and disease state [24,25]. Estrogens can upregulate EZH2 through estrogen receptor signaling in breast and endometrial tissues while androgens may also regulate EZH2 in different tissues such as the prostate [26,27].

EZH2 is highly associated with alterations in gene expression through the establishment of the H3K27me3 mark which creates a binding site for other proteins that further compact chromatin, making it less accessible for transcriptional machinery. In this way, it induces gene silencing, and affects crucial cellular processes including stem cell fate determination, cell differentiation, proliferation and tissue development [17].

EZH2 is an important regulator of self-renewal of embryonic stem cells as well as their pluripotency by repressing differentiation genes, thereby maintaining the stem cell state. During development, EZH2-mediated gene repression enables cell lineage specification by silencing improper genes for a given lineage while allowing for the expression of lineage-specific genes [28]. Nonetheless, PRC2 can additionally methylate protein substrates apart from histone, such as the zinc finger transcriptional factor GATA4, which is crucial for cardiac development and function, altering its transcriptional activity as well as its stability and turnover, thus modulating the overall levels of GATA4 in the cell [17]. However, EZH2 can also interact with non-histone or other proteins inducing gene repression via a PRC2-independent pathway. All these functional characteristics render EZH2 an important regulator of vital processes such as cell cycle progression, apoptosis, autophagy, DNA damage restoration, and aging-related cell damage [17]. EZH2 further promotes cell proliferation by repressing cyclin-dependent kinase inhibitors such as p16INK4a and p21CIP1, enabling cells to progress more readily through the cell cycle [29,30].

In normal cells, EZH2 helps maintain genomic stability and prevent inappropriate cell division, acting as a tumor suppressor [31]. It further plays a substantial role in cell fate determination and related signaling pathways [28]. It is also involved in maintaining the stem cell niches in various tissues by repressing differentiation genes, thereby ensuring a pool of undifferentiated stem cells for tissue repair and regeneration through balance regulation between stem cell renewal and differentiation [32].

The active involvement of EZH2 in a wide spectrum of biological processes justifies its implication in a broad array of diseases including cancer, and neurodegenerative disorders [17,22]. It is evident that this histone methyltransferase is equally involved in normal and pathological processes like immune and inflammatory responses, oncogenesis, cardiovascular diseases, and neurological disorders [22]. In the CNS, the physiological activity of EZH2 relies on proper neuronal function maintenance via the downregulation of genes responsible for nervous system disorders.

## 4. Role of EZH2 in Neuroinflammation

EZH2, being a multi-faceted and context-dependent regulator of immune response and neural cell function, plays a crucial role in neuroinflammation and associated diseases [10,12,13,22,33]. It has stimulated significant interest because of its complex involvement in maintaining microglial activation and regulation of proinflammatory cytokines expression in myeloid cells like monocytes/macrophages. Evidence of the involvement of EZH2 in CNS innate and adaptive immune reactions comes from studies where targeting of this molecule downregulated microglia activation and suppressed the induction of neuroinflammatory response [33]. Moreover, EZH2 has been further shown to support Treg stability and suppressive function as well as promote proliferation and survival of neural stem cells, exhibiting both a pro-inflammatory as well as an anti-inflammatory role depending on context.

### 4.1. Regulation of Inflammatory Gene Expression

The key mechanism of EZH2-regulated neuroinflammation is attributed to H3K27 methylation, resulting in transcriptional suppression [7]. This process is sequential, since lysine has the potential to become mono-, di-, or trimethylated (H3K27me3). Each one of these stages of methylation appears to fulfill a range of distinguished functions. H3K27me2, besides being the substrate for further trimethylation of H3K27, has been also shown to impede H3K27 acetylation, a process antagonistic to gene silencing that thrives in the lack of PRC2 [34].

**Figure 2:**
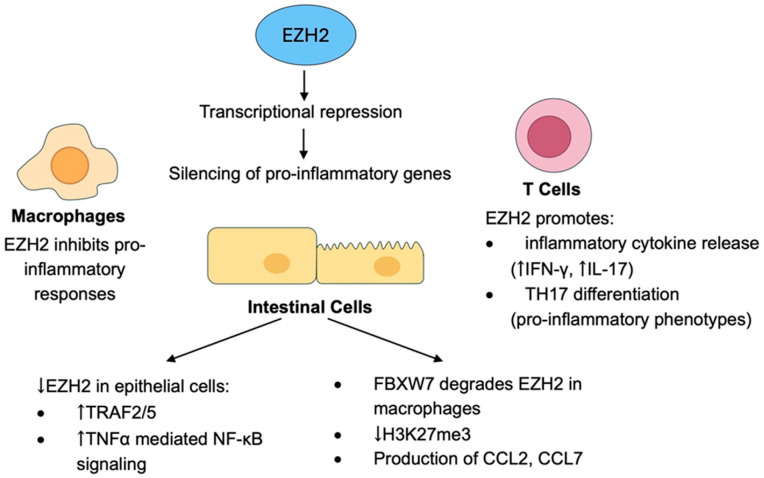
Role of EZH2 in the regulation of inflammatory gene expression.

By trimethylating H3K27, EZH2 can silence pro-inflammatory genes and pathways, exerting complicated functions in the myeloid lineage and blocking the proinflammatory responses of macrophages [35]. However, EZH2 can also regulate genes involved in immune suppression, promoting T cell activation and inflammatory cytokine release (e.g., IFN-γ, IL-17) [36]. It can drive the differentiation of T helper (Th) cells towards the pro-inflammatory Th17 phenotypes, relevant to autoimmune neuroinflammation (Figure 2) [37].

EZH2 was shown to have an impact on the preservation of the epithelial cell barrier in the context of inflammatory bowel disease (IBD). It was demonstrated that decreased EZH2 levels sensitize mice to experimental colitis, whereas overproduction of EZH2 in the intestinal epithelial cells reinforces their resistance to inflammatory conditions. The underlying mechanism suggests that under normal circumstances, the *TRAF2/5* genes are properly regulated, while in the case of diminished EZH2, the expression of these genes is increased, leading to activation of the TNFα-mediated NF-κΒ signaling. Consequently, EZH2 deficiency apart from contributing to deregulated inflammation, also compromises the protective function of NF-κΒ signaling by stimulating ITCH, an E3 ligase that impairs the c-FLIP protein (Figure 2) [38].

Additional studies in IBD have provided insight into the pro-inflammatory effects of EZH2 repression. The study of He et al. demonstrated that elevated E3 ligase FBXW7 expression in the intestine of IBD patients was highly associated with the severity of the disease. Conversely, diminished FBXW7 leads to downregulated expression of chemokines CCL2 and CCL7 by colonic CX3CR1hi resident macrophages and decreased accumulation of CX3CR1int pro-inflammatory mononuclear phagocytes in colitis tissue. By degrading EZH2 in macrophages, FBXW7 was found to diminish H3K27me3 and advance the production of CCL2 and CCL7 [39].

### 4.2. Microglia Activation

Macrophages are remarkably adaptive cells of the hematopoietic system that perform a wide range of functions during tissue damage or injury. Under these circumstances, tissue-located and peripherally derived macrophages are promptly activated and exert their pro-inflammatory actions, further triggering the evolution of inflammation-associated diseases. The CNS-resident macrophages are microglia and their role includes but is not limited to first-line protection of the brain against pathogens and CNS homeostasis during brain development [12]. Nonetheless, microglia have an essential role in the progression of neuroinflammatory disorders. The chronic activation of microglia reverses their protective potential, resulting in severe neurological damage and neurodegeneration [40].

**Figure 3:**
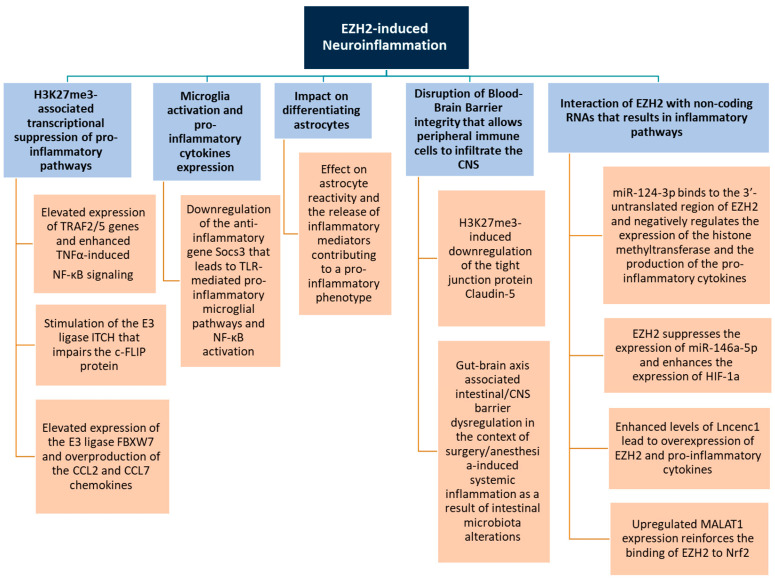
Main EZH2-induced mechanisms of neuroinflammation.

Studies have shown that EZH2 controls the inflammatory phenotype of microglia (Figure 3). The pro-inflammatory (M1-like) microglia express high levels of cytokines like IL-1β, IL-6, TNF-α, and EZH2 has been shown to promote this state by suppressing anti-inflammatory genes [41]. It is further considered as the mediator of microglia activation by directly targeting the anti-inflammatory gene *Socs3* [12]. Socs3 is activated by the Myeloid differentiation factor 88 (MyD88), eliciting the proteosome-related disintegration of TRAF6, a TNF receptor-associated factor that further activates NF-κΒ, and initiates inflammation [10]. The EZH2-related downregulation of *Socs3* induces the activation of TLR-mediated proinflammatory microglial pathways, leading to further activation of NF-κΒ and cytokine expression, increasing the inflammatory response [12].

Conversely, EZH2 has also been shown to mediate the suppression of excessive inflammation by repressing pro-inflammatory gene expression, especially in chronic settings [42]. During the recovery from injury or in response to IL-4, EZH2 can silence inflammatory genes, enabling a shift towards the anti-inflammatory M2-like phenotype [43]. The interaction of EZH2 with the TGF-β pathway or PI3K/Akt may also contribute to the resolution of inflammation and tissue repair.

Therefore, EZH2 can exhibit both pro- or anti-inflammatory effects depending on the duration and phase of activation. It may initially promote inflammation, but upon sustained activation, a feedback inhibition may take place which will suppress excessive inflammatory signaling, and eventually protect from tissue damage. Furthermore, a disease-specific function of EZH2 has been observed, depending both on the disease stage and microglial environment. More specifically, EZH2 has been shown to exacerbate demyelination in multiple sclerosis while it can support neuroprotection and repair in stroke recovery. Additional conditions such as hypoxia or oxidative stress and the cytokine milieu can further affect EZH2 activity. Some studies further suggest that there are sex differences associated with microglial activation, with females showing stronger immune responses in the CNS [44]. Since EZH2 regulates inflammatory gene expression, its levels or activity could also differ between males and females during neurodegenerative diseases like Alzheimer’s or multiple sclerosis. However, direct evidence for sex differences in EZH2 levels in neuroinflammation is still limited and an active area of research.

### 4.3. Astrocyte Function

Emerging studies shed light on the involvement of astrocytes in neuroinflammatory disorders, with both developing and mature astrocytes bearing the potential of being histone methylated [45,46]. There is evidence that EZH2 has an impact on differentiating astrocytes, yet there is no proof supporting its role in mature astrocytes [47] (Figure 3). It is possible that histone methylation prompts the production of inflammatory markers in astrocytes which function to exacerbate neuroinflammatory processes [48]. EZH2-mediated H3K27me3 can affect astrocyte reactivity and the release of inflammatory mediators. Dysregulation of EZH2 in astrocytes may lead to a pro-inflammatory phenotype, further driving neuroinflammation [48]. A study investigating the implication of EZH2 in rats with neuropathic pain demonstrated a regulatory role in spinal neuroinflammation. It was shown that H3K27-related astrocytic activation-induced overexpression of inflammatory mediators. This mechanism leads to excessive activation of post-injury spinal dorsal horn neurons which is a primary pathogenic factor of neuropathic pain [11].

EZH2-driven astrocytic changes play a significant and context-dependent role in shaping the clinical course of several neurological diseases. By modulating reactive astrogliosis through repression of genes such as *Socs3* or *NR4A2* which maintain the quiescent state, EZH2 can enhance astrocyte reactivity. This can be protective in the acute phase but chronic reactivity can limit neuronal regeneration and exacerbate functional deficits [12,49,50]. Therefore during spinal cord injury or stroke, EZH2-induced overactivation of astrocytes will initially protect but subsequently, it will hinder axonal regrowth, and delay functional recovery [51]. Additionally, EZH2 regulates the secretion of inflammatory cytokines and chemokines such as IL-6 and CCL2, affecting microglial activation and T-cell infiltration [22]. In this way, EZH2 derived from astrocytes can sustain inflammation and dysfunction of BBB, contributing to faster cognitive decline [52].

By silencing genes encoding neurotrophic factors (such as BDNF, GDNF), EZH2 can compromise the ability of astrocytes to support neurons and maintain synaptic plasticity [48]. This can be of particular importance for Parkinson’s disease, since reduced neurotrophic support may accelerate dopaminergic neuron loss and worsen motor symptoms.

EZH2 can also affect the astrocyte-neuron crosstalk and potentially contribute to excitotoxicity or synaptic dysfunction [48,53] which are particularly important in epilepsy or autism spectrum disorders.

It is therefore evident that astrocytic changes mediated by EZH2 can act as epigenetic switches that modulate inflammation, neuroprotection, and regeneration. They depend on timing and disease stage and they can improve or deteriorate the clinical outcome of neurological diseases.

### 4.4. Blood–Brain Barrier Integrity

EZH2’s role in blood–brain barrier (BBB) integrity is not uniform but depends on the microenvironmental cues, disease context and the specific cell types involved such as endothelial cells, astrocytes, and microglia (Figure 3).

Although mainly disruptive, there is evidence that EZH2 may also be involved in the maintenance of the BBB integrity, at the early stages of Alzheimer’s disease through regulation of genes implicated in BBB development and maintenance. Specifically, EZH2 contributes to the regulation of genes involved in endothelial cell function and BBB stability while impaired EZH2 function was shown to compromise BBB integrity, facilitating neuroinflammation [54,55,56]. In this way, chronic inflammation observed in later stages of AD partly mediated by EZH2, may indirectly impair BBB function.

Neuroinflammation is often associated with disruption of the BBB, allowing peripheral immune cells to infiltrate the CNS. A distinctive case of BBB breakdown as a result of the underlying neurovascular impairment is that of major depressive disorder (MDD), with extensive research suggesting that this specific mechanism is fundamental for the pathogenesis of the disease. Tight junction (TJ) proteins (claudin-5, occludin) are essential regulators of BBB structural and functional integrity and homeostasis, regulated by H3K27me3 histone mark established by EZH2 [54]. In individuals with depression and stress, claudin-5, the most prevalent TJ protein is diminished, and BBB permeability is increased [54]. Taking into consideration the significance of inflammation as a contributing factor in the pathology of MDD, the study of Sun et al. demonstrates the role of BBB in preventing the transfer of peripherally derived pro-inflammatory molecules into the brain. Their results showed that H3K27me3 stimulates the downregulation of claudin-5, contributing to the initiation of the disease, while attenuation of EZH2 was shown to impede the suppression of claudin-5, restore the BBB dysfunction and overturn the depressive symptoms [54].

BBB disruption can also occur through surgery and anesthesia, leading to perioperative neurocognitive disorders (PND), particularly in the elderly. A principal characteristic of PND is hippocampal neuroinflammation, as a result of the surgical trauma that stimulates acute systemic inflammatory responses. EZH2 plays a pivotal role in this inflammatory mechanism that results in NF-κΒ-associated cytokines expression, including IL-6, IL-1β, and TNF-α [57]. Additional data shed light on the role of neuroinflammation-associated BBB disruption on postoperative cognitive dysfunction (POCD), especially in individuals with Alzheimer’s Disease [55]. This is a CNS complication with clinical manifestations including loss of memory, orientation disorder, and declined social activity. The study showed that neuroinflammation and BBB dysfunction are the major contributors to the disease, suggesting that intestinal microbiota alterations could initiate systemic inflammation that further disturbs the intestinal and CNS barrier through the gut–brain axis, and further indicating that prebiotics can be used as a potential preventative intervention [55].

### 4.5. Interaction with Non-Coding RNAs

EZH2 can interact with various non-coding RNAs, such as microRNAs (miRNAs) and long non-coding RNAs (lncRNAs), which can modulate its activity and influence inflammatory pathways (Figure 3). These interactions add another layer of regulation to EZH2’s role in neuroinflammation [58,59]. A recent study elucidates the effect of EZH2 interaction with non-coding RNAs on the pathogenesis of neuroinflammation and neuropathic pain [22]. Accordingly, microRNA-124-3p (miR-124-3p), a member of the miRNA family, has inhibitory effects on EZH2, thus negatively regulating the pro-inflammatory cytokines expression and the progression of neuropathic pain. It has been shown that miR-124-3p binds to the 3′-untranslated region of EZH2, leading to decreased protein expression of the histone methyltransferase and alleviation of the symptoms. Another miRNA implicated in the neuroinflammatory responses of neuropathic pain is miR-146a-5p, regulated by EZH2. In particular, EZH2 suppresses the production of miR-146a-5p resulting in enhanced expression of the transcriptional factor HIF-1a that acts as a positive modulator of inflammation, thus contributing to aggravated neuroinflammatory symptoms.

LncRNA embryonic stem cell expressed 1 (Lncenc1) interferes with signaling pathways in terms of neuroinflammatory regulation. Neuropathic pain leads to increased expression of Lncenc1, which subsequently results in overexpression of EZH2 and proinflammatory cytokines. Another lncRNA that seems to relate to the pathogenetic mechanisms of neuropathic pain is the metastasis-associated lung adenocarcinoma transcript 1 (MALAT1). It has been demonstrated that MALAT1 binds to EZH2 and upregulated MALAT1 production improves the EZH2 function by enhancing its binding to the target gene, the nuclear factor erythroid 2-related factor 2 (Nrf2), which moderates the neuroinflammatory symptoms [22].

## 5. EZH2 Role in Neuroinflammatory Diseases

Clinical data demonstrate the strong association between EZH2 and a wide spectrum of neuroinflammatory conditions, with aging presenting a key primer factor in lowering EZH2 control [60]. A global decrease in EZH2 mRNA and protein expression has been detected with aging in stem cells, immune cells, brain, and vascular tissues, amplifying the magnitude and impact of EZH2 dysregulation during disease. A decline of EZH2 levels in neurons and astrocytes has been shown to increase the neuronal vulnerability to neuroinflammation and reduce their plasticity. Subsequently, reduced H3K27me3 levels lead to chromatin “de-repression” inducing more transcriptional noise and increased inflammatory gene expression [10].

Although the overall EZH2 levels may decrease with age, certain stimuli related to diseases such as neuroinflammation, brain tumors or acute injury of the aged brain may induce EZH2 expression, especially in astrocytes and microglia, increasing its levels locally. Moreover, exhausted T cells during aging have been shown to exhibit increased EZH2 levels to maintain a dysfunctional phenotype, indicating the tissue- and context-specific role of EZH2 [36]. Additionally, epigenetic feedback loops and inflammatory cytokine exposure (IL-6, TNF-α) may upregulate EZH2 levels.

Aberrant EZH2 activity has been associated with the onset and progression of neurodegenerative disorders, such as AD or PD, which may reflect both age and disease state [1,61]. However, the effects of EZH2 are not limited to neurodegeneration, but also extend to other neurological conditions such as depression, as demonstrated by studies on depression-like experimental models [61]. Additionally, EZH2 was shown to be involved in the generation of neuropathic pain, with the levels of H3K27me3 and EZH2-microglia being considerably elevated after nerve injury as indicated by animal experiments [7]. Notwithstanding, the functions of EZH2 have been reported in a much wider range of disorders including inflammatory bowel disease [62], or even the pathogenesis of subarachnoid hemorrhage [63] and ischemic stroke [64].

Keeping in mind that neuroinflammatory diseases are highly heterogeneous ranging from acute sterile inflammation to autoimmune, infectious and chronic neurogenerative conditions, EZH2 functions are context-dependent and not considered universally harmful or helpful.

### 5.1. Multiple Sclerosis (MS)

In MS, autoimmune T cells attack the myelin sheath, leading to neuroinflammation and demyelination. EZH2 has been shown to promote Th17 cell differentiation, which is a key pathogenic T cell subset in MS [65]. Th17 cells produce pro-inflammatory cytokines such as IL-17 and IL-22, driving the immune response against myelin and triggering inflammation [66]. On the other hand, EZH2 is also involved in the differentiation of regulatory T cells (Tregs), which are responsible for immune suppression [67]. In MS, the balance between Th17 and Treg cells is critical, and modulating EZH2 activity can influence the disease course by affecting the balance between pro-inflammatory and anti-inflammatory responses [12,68]. Thus, EZH2 inhibition may shift the balance in favor of Tregs, leading to reduced autoimmunity.

A recent study has further shown that EZH2 leads to proinflammatory gene expression mediated by toll-like receptors (TLR). The underlying mechanism of EZH2 deficiency decreases macrophage and microglial activation and subsequently alleviates the MS-related autoimmune neuroinflammatory symptoms. It has been shown that EZH2 reduction directly triggers the production of the anti-inflammatory mediator Socs3, resulting in Lys88-associated TRAF6 ubiquitination and degradation, further inducing the repression of TLR-related MyD88-dependent NF-κΒ activation and the silencing of genes associated with macrophage–microglial activity and autoimmune neuroinflammation. The aggravating role of EZH2 in MS is also confirmed by the increased predisposition of MS patients’ brain tissue to express EZH2 as opposed to healthy individuals [12].

Additionally, EZH2 may play a key role in remyelination in the CNS since it can influence the differentiation of oligodendrocyte precursor cells (OPCs) into mature oligodendrocytes, which are required for myelin repair [68]. Loss of EZH2 in oligodendrocytes and OPCs impairs remyelination and leads to more permanent damage in MS lesions. EZH2 activation in the early phase of MS could therefore support myelin repair, whereas EZH2 inhibition in the late phase might disrupt chronic inflammation but could also impair tissue repair and exacerbate long-term disability.

### 5.2. Alzheimer’s Disease (AD)

In AD, microglial cells become chronically activated in response to amyloid-beta (Aβ) plaques and EZH2 has been shown to play a crucial role in regulating this microglial activation. The EZH2-mediated repression of pro-inflammatory genes in microglia may prevent excessive inflammation in the brain, suggesting that EZH2 can suppress the inflammatory response to Aβ deposition [41].

However, loss of EZH2 in microglia has been associated with exaggerated inflammatory responses and worsened pathology, as microglial cells may become overly reactive and contribute to neurodegeneration [54,55]. A novel study investigating the impact of lncRNA XIST in AD through the epigenetic modulation of Aβ-degrading enzymes’ expression demonstrated that AD individuals exhibit elevated lncRNA XIST levels in conjunction with noticeable signs of neuroinflammation. The outcome of the study revealed that lncRNA XIST downregulates the production of the Aβ-degrading enzyme neprilysin (NEP) through epigenetic modulation by binding to EZH2. In particular, by epigenetically silencing NEP in AD individuals following interaction with EZH2, lncRNA XIST contributes to Aβ accumulation and aggravation of Aβ-induced neuroinflammatory lesions. Conversely, suppression of lncRNA XIST attenuates the Aβ-associated neuroinflammatory state and limits the H3K27me3 as well as the EZH2 recruitment of the NEP promoter region [69,70].

Additionally, EZH2 can indirectly affect the clearance of amyloid plaques by modulating the microglial phenotype. Microglia can either adopt a pro-inflammatory (M1-like) or anti-inflammatory (M2-like) state, influencing their phagocytic and clearance ability [71]. EZH2 can further promote the M2-like microglial state, which supports Aβ clearance and neuroprotection, indicating that inhibition of EZH2 may prevent this switch, thereby impairing Aβ clearance [33].

Furthermore, EZH2 plays a key role in neurogenesis, particularly in the hippocampus, an area significantly affected by AD [72,73]. In aging and AD, reduced EZH2 expression in neural stem cells (NSCs) was shown to lead to impaired neurogenesis [47,74]. This can exacerbate cognitive decline by limiting the brain’s ability to generate new neurons to replace those lost to AD.

### 5.3. Parkinson’s Disease (PD)

In PD, the progressive degeneration of dopaminergic neurons in the substantia nigra is accompanied by chronic microglial activation. EZH2 regulates the activation of microglia, and its loss leads to uncontrolled microglial activation, which is harmful to dopaminergic neurons [22,41].

Emerging studies reveal that the EZH2-induced *Socs3* gene inactivation contributes to unregulated NF-κΒ function and pro-inflammatory cytokines production also within the framework of PD. The overexpression of H3K27me3 in the brain of PD patients confirms the aggravating involvement of EZH2 in the pathogenesis of the disease. Abnormal EZH2-associated H3K27me3 leads to the silencing of hallmark PD genes, including the α-synuclein in the substantia nigra neurons of the patients [75]. A study by Cai et al. provides insight into the mechanism underlying the lncRNA metastasis-associated lung adenocarcinoma transcript 1 (lncRNA MALAT1)-induced PD neuroinflammation and reveals its elevated expression in the context of the disease [76]. MALAT1 triggers neuroinflammatory responses by enriching EZH2 binding to the promoter of *NRF2* and epigenetically represses its expression. Downregulation of MALAT1 attenuates the overexpression of NRF2 and the subsequent activation of inflammasome as well as the production of reactive oxygen species (ROS), associated with PD initiation and progression. The abovementioned study was conducted in both PD mice and microglial cell models and the effects of MALAT1 in microglia were equally established, suggesting that it mitigates NRF2 production by enriching the recruitment of EZH2 to the *NRF2* promoter, which successively elevates ROS production. PD and other neurodegenerative diseases can originate from the chronic neuroinflammation induced by microglial activation. Normally, activated microglia are neurotoxic to contiguous neurons, which, in turn, trigger supplementary activation of microglia. This vicious circle contributes to escalating neuroinflammatory responses and ongoing neuronal damage and loss (Table 1) [76,77].

EZH2 may help to reduce microglial neurotoxic effects by promoting the anti-inflammatory (M2-like) phenotype, thereby offering neuroprotection to dopaminergic neurons. EZH2-mediated repression of inflammatory pathways in dopaminergic neurons is considered to be neuroprotective, as the over-activation of inflammatory genes in these neurons can contribute to cell death [78].

Conversely, the activation of EZH2 may also play a protective role in dopaminergic cell survival, especially in models of PD where neuronal apoptosis is accelerated due to excessive inflammation. EZH2 also impacts the regulation of autophagy and mitophagy pathways, which are essential for maintaining mitochondrial health. Impaired mitophagy in PD leads to mitochondrial dysfunction and neuronal death. EZH2, by regulating autophagic genes, could contribute to mitochondrial turnover and help mitigate neurodegeneration [77,78].

Following these studies, it is evident that EZH2 inhibition in neurological diseases can be a promising therapeutic approach with beneficial outcomes for certain conditions, bearing in mind that its effects are highly stage- and context-dependent. In respect to stage, during the active demyelinating phase in MS, EZH2 can suppress the activation of pro-inflammatory glial cells, and thus reduce immune infiltration as well as the lesion burden [68]. In a similar way, at the early neurodegenerative phase of PD, glial EZH2 activity may exert a protective role in the dopaminergic neurons through the mitigation of neuroinflammation [77,78].

Additionally, in the acute and subacute phases of ischemic stroke, EZH2 may be involved in the reduction in BBB disruption, decreasing neuroinflammation and infarct volume, possibly leading to neuroprotection [33,79]. Likewise, at the early to intermediate phase of traumatic brain injury, it is implicated in limiting the activation of astrocytes and microglia, preserving the integrity of BBB and preventing secondary damage [43,51]. In cancer context, during glioblastoma progression, EZH2 inhibition has been shown to reduce tumor cell proliferation, inhibiting abnormal angiogenesis and immunosuppressive signaling [80]. Overall, early stages of neurodegeneration and glial overactivation, acute inflammation and high-grade gliomas seem to present the best conditions for applying EZH2 inhibition strategies.

## 6. Impact of EZH2 in Cholinergic Neurotransmission in Neuroinflammation

The cholinergic anti-inflammatory pathway (CAP) is a major neuroimmune mechanism where acetylcholine (ACh)—released mainly via the vagus nerve—suppresses peripheral inflammatory responses. ACh acts primarily on α7 nicotinic acetylcholine receptors (α7nAChR) on immune cells (like monocytes, macrophages, and lymphocytes) to inhibit pro-inflammatory cytokine release (e.g., TNF-α, IL-1β). EZH2 in immune cells such as monocytes, and lymphocytes, normally helps to regulate gene expression by silencing pro-inflammatory genes via H3K27 trimethylation (H3K27me3) [81,82].

After ischemic stroke, this pathway can become dysregulated and the reduced cholinergic tone heightens systemic inflammation (“stroke-induced immune dysfunction”) affecting not only the brain but also nucleated blood cells (peripheral immune system) [83]. Systemic inflammation (in the blood) leads to dynamic changes in EZH2 levels. Studies suggest that EZH2 expression is often reduced in activated monocytes/macrophages during acute inflammation, allowing pro-inflammatory genes to be expressed. Conversely, in some conditions (especially in regulatory T cells or exhausted immune cells), EZH2 can be upregulated to promote immunosuppressive or tolerance phenotypes [81,84].

Upon pharmacological enhancement of cholinergic tone (e.g., using acetylcholinesterase inhibitors like donepezil, galantamine, or direct α7nAChR agonists), the α7nAChR is activated in immune cells, suppressing NF-κB signaling, and reducing pro-inflammatory cytokine expression (like IL-1β, TNF-α), potential reactivating the EZH2 expression, re-establishing H3K27me3-mediated gene repression of inflammatory genes.

In rodent models of ischemic stroke, vagus nerve stimulation (a method to boost cholinergic anti-inflammatory signaling) reduced systemic inflammation and improved neurological outcomes while immune cells showed increased markers of chromatin repression (histone modifications like H3K27me3) [85].

Therefore, boosting cholinergic neurotransmission after ischemic stroke likely modulates EZH2 expression/activity in nucleated blood cells, promoting an anti-inflammatory epigenetic program and helping to resolve harmful systemic inflammation. This makes EZH2 a fascinating epigenetic link between neuroimmune therapies and peripheral immune responses post-stroke.

## 7. EZH2 Targeting Options in Treatment Strategies

In the last few years, the development of EZH2 inhibitors has emerged as a promising area of investigation for the therapeutic approach to various diseases [7,10,22,33,75,86]. Given the increased heterogeneity of neuroinflammatory diseases, EZH2 inhibition cannot be inherently “anti-inflammatory” or “pro-repair” but rather its effects depend on the disease type and stage, the specific inflammatory cells involved, the tissue repair needs as well as patient age and comorbidities. Although EZH2 inhibition in neuroinflammatory or neurodegenerative diseases is still experimental, it is worth looking at the current available options.

EZH2 inhibitors are categorized into indirect and direct types. Indirect EZH2 inhibitors are also known as S-adenosyl-L-homocysteine (SAH) hydrolase inhibitors. The universal methyl donor S-adenosyl-L-methionine (SAM) transfers the methyl group to the lysine side chain of the protein acceptor during the synthesis of H3K27 methylation in a catalytic way. When a methyl group is removed, SAM turns into SAH (S-adenosyl-L-homocysteine) and SAH hydrolase is the enzyme that further metabolizes it. EZH2 indirect inhibitors function by downregulating the SAH hydrolase activity, resulting in SAH being built up in cells. Thus, the methyl group is prevented from being released from SAM, resulting in indirect inhibition of the EZH2 methyltransferase activity. The category of indirect EZH2 inhibitors includes DZNep (3-deazaneplanocin A) and D9, an analog of DZNep, but with less toxic effects. DZNep is a non-selective EZH2 antagonist, which means that it impedes all histone methylation and inhibits various methyltransferase activities [22].

The latest class of highly effective EZH2 inhibitors are the direct inhibitors, which compete with the SAM binding site of the EZH2 SET domain. Accordingly, the direct inhibitors also referred to as SAM-competitive inhibitors, include UNC1999, EI1, GSK126, GSK503, EPZ005687, and EPZ-6438, amongst others [22]. GSK126 is a potent EZH2 inhibitor but poorly crosses the BBB and high doses are needed which increase the risk of systemic side effects [87].

The neuroprotective effects of DZNep after ischemic brain injury have been the subject of discussion in several studies [33,88]. There is strong evidence that EZH2 expression is elevated in microglia in the context of ischemic stroke, further contributing to its pathophysiology and the accompanied neurological impairments, presumably by activating STAT3 (Signal Transducer and Activator of transcription 3). Pro-inflammatory cytokine production with microglia activation leads to unsuccessful post-stroke outcomes. Taking this into consideration, the regulation of microglia activation can lay the foundations for innovative ischemic stroke therapeutic approaches. Pharmacologically, DZNep stimulates the reduction in EZH2 and reverses the H3K27me3 accumulation, making it an ideal therapeutic molecule for a wide range of pathologies, including ischemic stroke [33]. The neuroprotective effects of DZNep were shown in mice with experimental stroke. The administered EZH2 inhibitor reduced microglia’s pro-inflammatory (CD86+) activation and the pro-inflammatory cytokines IL-1β, IL-6, TNF-α, and CXCL10. Furthermore, it restricted the phosphorylation of STAT3 and stimulated microglia’s anti-inflammatory (CD206+) polarization [33].

Moreover, as the role of EZH2 in neuropathic pain has already been established, targeting this molecule could alleviate neuroinflammation leading to analgesic effects. EZH2 regulates nerve injury-induced neuropathic pain through microglia activation and proinflammatory cytokines and chemokines production. H3K27 methylation and anti-inflammatory genes downregulation were the factors contributing to the pro-inflammatory activity of EZH2. These genes are considered anti-inflammatory mediators as they negatively regulate the induction of neuropathic pain. Thus, suppression of these genes results in extreme stimulation of neurons implicated in the pain pathway. DZNep and GSK-126 were shown to exert better outcomes in neuropathic pain by inhibiting EZH2 (Table 2) [22]. The administration of these inhibitors in rats with partial sciatic nerve ligation-related neuropathic pain demonstrated both their role in the treatment and prevention of thermal hyperalgesia and mechanical allodynia. Furthermore, microglia and astrocyte activation along with pro-inflammatory mediators’ expression were equally mitigated, while EZH2 and H3K27me3 levels were normalized. GSK126 administration led to relevant results in the context of chronic constriction injury associated with neuropathic pain, as well as in cases of brachial plexus avulsion [22].

Another EZH2 inhibitor, EPZ-6438, also known as tazemetostat, has been shown to downregulate the expression of inflammatory mediators in neuropathic pain (Table 2) [86]. This competitive inhibitor suppresses the production of several inflammatory mediators, namely signal transducer and activator of transcription 1 (STAT1) and interferon regulatory factor 1 (IRF1). Although tazemetostat is generally well tolerated, it may cause secondary malignancies due to epigenetic dysregulation and has a limited BBB penetration making it not ideal for neuroinflammation [89]. It has been demonstrated that depressive behaviors and aging-related behavioral deficits, conditions with increased levels of EZH2 and H3K27me3 in animal models, can be attenuated with the administration of EPZ-6438 [10]. Furthermore, EZH2 and H3K27me3 are highly involved in the pathophysiology of neuroinflammatory-related early brain injury after subarachnoid hemorrhage, explaining why administration of EPZ-6438 remarkably ameliorated the neurological deficit consequences. The underlying mechanism of this process mainly relies on Socs3 activation, with TRAF6 proteins, NF-κΒ, p65, and pro-inflammatory cytokines being also reduced [63].

Additional direct inhibitors competing with SAM to attenuate EZH2 methyltransferase function are GSK-343 and GSK-926 (Table 2). By diminishing H3K27me3 levels, these inhibitors present a hopeful therapeutic prospect for managing brain aging and the associated progression of cognitive decline [7]. Regarding the pathogenesis and the course of neurodegenerative disorders, focusing on the example of Parkinson’s disease, the role of epigenetic modulators, including EZH2, has been significantly highlighted. In this respect, GSK-343 has been regarded as an EZH2 inhibitor with neuroprotective properties against dopaminergic degeneration, resulting in refined behavioral deficit outcomes and modification of the disease manifestations. Moreover, GSK-343, by regulating the NF-κB/IκBα pathway, the secretion of cytokines, and the stimulation of glia, seems to mitigate neuroinflammation, indicating that this inhibitor could be evaluated as an effective therapeutic alternative for Parkinson’s disease [75].

Another promising inhibitor of EZH2 is the polyphenol curcumin, derived from the *Curcuma longa* plant, referred to as turmeric (Table 2). Curcumin is recognized for its anticancer, antioxidant, and anti-inflammatory functions. It regulates miRNAs, represses the expression of DNA methyltransferases, and is particularly involved in the regulation of histone methylation, making it a potential EZH2 inhibitor for managing a wide range of neurological disorders [7].

Nonetheless, it is essential to address potential limitations and off-target effects with the aim of ensuring a safe and effective clinical translation of EZH2 inhibitors. It has to be noted that EZH2 is vital for normal cell function and its systemic inhibition may cause unwanted effects like impaired immune defense, bone marrow suppression, and toxicity in healthy tissues. Also, many EZH2 inhibitors are not perfectly specific and can inhibit other methyltransferases or unrelated proteins. To this end, an experimental model study assessing DZNep side effects and potential safety challenges on multiple organs detected a reversible splenomegaly, prolonged testis reduction and erythropoiesis, without, however, any effect in mice behavior [90].

Considering some limitations of the EZH2 inhibitors, like the low bioavailability, the questionable effectiveness, the high molecular weight, and the large dosages needed, a recent study introduced a new series of 3-acrylamido-2-methyl-N-((2-oxo-1,2-dihydropyridin-3-yl) methyl) benzamide derivatives as covalent EZH2 inhibitors, with SKLB-03176 appearing as the most promising inhibitory agent. It covalently bound to Cys663 at the SAM site of EZH2 and exerted minor off-target effects, without presenting toxicity to normal cells. The covalent binding ensures a prolonged pharmacodynamic duration, further enabling lower effective dosages. It is worth mentioning that SKLB-03176 had also the potential to inhibit a range of EZH2 mutations. It is important for future research to explore the prospects of introducing covalent EZH2 inhibitors as a potential therapeutic approach for neuroinflammatory diseases [91].

Additional challenges for EZH2 inhibitors remain the poor BBB penetration in some cases as well as the dosage and specific timing of administration, since inflammation is composed of early and late phases, and EZH2 inhibition at the wrong time could block the necessary immune responses (early phase) or impair repair (late phase). Furthermore, the heterogeneity of target cells needs to be considered since EZH2 plays very different roles in different cells (such as in microglia vs. T cells vs. neurons).

**Table 2:** EZH2 targeting options in neuroinflammation-related conditions.

EZH2 Inhibitor/Chemical Compound	Type of Inhibitor	Mechanism of Action/Effect	Disease	Type of Study	Side-Effects	Reference
DZNep/ 3-Deazaneplanocin A	Indirect	- Reduction in microglial pro-inflammatory activation (CD86+) and pro-inflammatory cytokines IL-6, IL-1β, TNF-α, and CXCL10 - Restriction of STAT3 phosphorylation - Stimulation of microglial anti-inflammatory polarization (CD206+) - Reduction in EZH2 activity and reversal of H3K27me3 accumulation	Ischemic stroke	In vitro and in vivo		[33]
			Neuropathic pain	In vivo		[22]
					- Potential neurotoxicity, impaired neurogenesis, BBB disruption - Anemia, immune suppression in animal models - Poor clinical viability due to off-target effects and global methylation interference	
GSK-126	Direct	- Inhibition of microglial, astrocytic, and pro-inflammatory mediators’ expression, - Standardization of EZH2 and H3K27me3 levels	Neuropathic pain	In vivo	- Nausea, diarrhea, reduced appetite - Mild anemia, neutropenia, lymphopenia - Changes in T cell function or inflammatory cytokines that can be beneficial or adverse due to disease context	[22]
EPZ-6438/Tazemetostat	Direct	- Inhibition of inflammatory mediators’ expression, including IRF1 and STAT1 - Socs3 activation - Reduction in TRAF6 proteins, NF-κΒ, p65, and pro-inflammatory cytokines production	Neuropathic pain	In vitro	Nausea, constipation, anorexia	[86]
			Depressive behaviors	In vivo	Anemia, neutropenia, thrombocytopenia	[10]
			Aging-related behavioral deficits	In vivo	Mild transaminase elevations	[10]
			Subarachnoid hemorrhage brain injury	In vivo	Fatigue, pain, peripheral edema, weight loss	[63]
GSK-343	Direct	Neuroprotective properties against dopaminergic degeneration through regulation of the NF-κB/IκBα pathway, the production of cytokines, and the activation of glia	Brain aging-related cognitive decline	In vitro	Cytopenias, impaired stem cell function	[7,92]
			Parkinson’s disease	In vivo	Not characterized (poor oral bioavailability limits systemic exposure) - Altered T-cell activity, microglial cytokine imbalance	[75]
Curcumin	-	Anticancer, antioxidant, and anti-inflammatory properties, regulation of histone methylation	Neuroinflammation-related neurological disorders	In vitro	Nausea, diarrhea, bloating, indigestion (in high doses) - Reduced iron absorption - Elevated liver enzymes (rare) - Skin rash, itching (rare)	[7,93]

Selecting an appropriate EZH2 inhibitor for neurological diseases requires careful consideration of BBB penetration, target specificity, disease stage and severity. Regarding BBB penetration, there is a limitation of data because most EZH2 inhibitors were developed for oncology and may not naturally cross the BBB. UNC1999 being a dual EZH2/EZH1 inhibitor, has shown the best BBB penetration in some CNS models [94], followed by tazemetostat which exhibits low to moderate BBB potential and GSK126 with poor and limited CNS use [10,22,63,86].

With respect to target specificity, EZH2 can often share functions with EZH1, possibly exerting unwanted epigenetic or immune effects due to off-target inhibition. Therefore, EZH2-selective inhibitors such as GSK126 can be selected for acute inflammatory suppression (like in stroke or acute MS relapse) to avoid long-term epigenetic disruption while dual inhibitors (such as UNC1999) may be more applicable in chronic AD or glioblastoma, providing a broader control of epigenetic reprogramming. Regarding stage-specific effects, EZH2 inhibitors are generally regarded as anti-inflammatory in the acute phase but possibly anti-repair in the chronic phase and therefore caution is demanded. Therefore, for the acute phase of stroke and TBI (<72 h) as well as in the early neuroinflammation stage of AD and PD, an inhibitor that is fast-acting, BBB-penetrant and EZH2-selective is recommended. In MS remission and late or chronic degeneration, EZH2 inhibitors should be avoided or used in lower doses with caution and combined with neurotrophic agents.

Current research efforts are directed to the generation of alternative drug modalities for EZH2 targeting to improve the specificity and overcome resistance in complex tissues. Antisense oligonucleotides that bind EZH2 mRNA hold greater promise since they enable degradation via RNase H or inhibition of translation [95].

Specifically, the IONIS-EZH2Rx has demonstrated mRNA knockdown in preclinical cancer models while other antisense oligonucleotides have shown effectiveness in CNS-targeted drugs such as nusinersen for spinal muscular atrophy. siRNAs inducing EZH2 mRNA degradation and miRNA mimics restoring the expression of EZH2-targeting microRNAs (such as miR-26a and miR-101), can also be suitable for CNS diseases and are currently explored preclinically. Proteolysis targeting chimeras (PROTACs) of EZH2, like MS1943, are further investigated in preclinical settings, exhibiting substantial activity in tumor models with EZH2 mutations; however, their BBB penetration remains a challenge [96].

To overcome these problems, antibody–drug conjugates (ADCs), liposomes, or exosomes can be used to deliver EZH2 inhibitors specifically to inflamed brain regions or immune cells, or administered together with anti-inflammatories, neuroprotectants, or cholinergic agents to balance risks. Although primarily used to deliver EZH2 inhibitors in tumors, ADCs against glial fibrillary acidic protein (GFAP), CD11b, or translocator protein (TSPO) markers may be used to target brain-resident cells in neurological diseases. Recently, engineered ADCs that can cross the BBB using ligands to insulin receptors or transferrin have been generated. A more promising strategy focuses on the liposomal formulations of hydrophobic EZH2 inhibitors such as GSK126 and GSK343 aiming to improve their stability and time of circulation as well as off-target effects. In order to facilitate BBB penetration, PEGylation can be applied for surface modification as well as several ligands such as transferrin and RVG peptides. Recent studies have explored intranasal liposomal delivery of EZH2 inhibitors in multiple sclerosis, AD and gliomas [97]. However, the most promising approach for CNS diseases is the use of exosomes because of their natural BBB penetration, low immunogenicity and biodegradation. Exosomes loaded with antisense oligonucleotides or siRNA as well as EZH2 inhibitors can target EZH2 mRNA and they can be engineered to express surface proteins to specifically target astrocytes, microglia or neurons.

## 8. Conclusions

Taken all together, there is an evident association between histone methyltransferase EZH2 and regulation of neuroinflammation through canonical (H3K27me3-dependent) and non-canonical mechanisms, affecting microglia, astrocytes, as well as neurons. The multiple functional roles of EZH2 prompt us to consider it as a versatile epigenetic modifier, whose functions must be analyzed contextually in a cell type and disease stage-specific manner. Most studies reveal that inhibition of EZH2 is more beneficial in chronic inflammation rather than in early development or repair phases. Therefore, EZH2 inhibition must be carefully evaluated to avoid the risk of broad-spectrum effects.

In comparison to other repressive epigenetic regulators such as HDAC and DNA methyltransferases (DNMTs), EZH2 is considered more specific in targeting inflammatory, and developmental genes through H3K27me3 pathways than HDAC which can affect many genes simultaneously (especially immediate-early inflammatory genes), or DNMTs which exert even more broad and irreversible long-term repression of inflammatory mediators and anti-inflammatory genes.

In our opinion, EZH2 targeting with small-molecule inhibitors or antisense oligonucleotides, offers a more precise and flexible way to modulate neuroinflammation. Additional studies are warranted to elucidate the mechanisms underlying the therapeutic effects of already existing EZH2 inhibitors as well as evaluate their safety and efficacy in clinical settings. As we move forward, extensive research that focuses on brain-penetrant drugs, cell-specific delivery, and careful timing to avoid blocking beneficial immune responses, minimizing toxicity and off-target gene effects is required to improve the lives of individuals affected by these debilitating conditions.

## Figures and Tables

**Table 1:** Pro- and anti-inflammatory roles of EZH2 in neurodegenerative diseases.

Disease	Pro-Inflammatory	Anti-Inflammatory/Repair	Reference
Multiple Sclerosis	EZH2 promotes Th17 differentiation, exacerbates autoimmunity	EZH2 promotes Treg stability; supports remyelination in oligodendrocytes	[65,66,67,68]
Alzheimer’s Disease	Loss of EZH2 enables chronic microglial activation; worsened neurodegeneration	EZH2 supports Aβ clearance; promotes microglial polarization to M2-like state	[41,47,54,55,72,73,74]
Parkinson’s Disease	Excessive EZH2 inhibition induces uncontrolled microglial activation	EZH2 induces neuroprotective effects in dopaminergic neurons; regulation of autophagy/mitophagy	[41,75,76,77,78]
